# Carbon Nanotubes Modified With Au for Electrochemical Detection of Prostate Specific Antigen: Effect of Au Nanoparticle Size Distribution

**DOI:** 10.3389/fchem.2019.00147

**Published:** 2019-03-27

**Authors:** Andrés Felipe Quintero-Jaime, Ángel Berenguer-Murcia, Diego Cazorla-Amorós, Emilia Morallón

**Affiliations:** ^1^Departamento de Química Física and Instituto Universitario de Materiales de Alicante (IUMA), University of Alicante, Alicante, Spain; ^2^Departamento de Química Inorgánica and Instituto Universitario de Materiales de Alicante (IUMA), University of Alicante, Alicante, Spain

**Keywords:** carbon nanotubes, PSA detection, chronoamperometry, gold nanoparticles, immunosensor

## Abstract

Different functionalized Multi-Wall Carbon Nanotube and gold nanoparticles (AuNPs) were synthesized as biosensor electrodes. These materials have been applied to the detection of the Prostate Specific Antigen (PSA). The synthesis of AuNPs was carried out using polyvinylpyrrolidone (PVP) as protecting agent. The PVP/Au molar ratio (0.5 and 50) controls the nanoparticle size distribution, obtaining a wide and narrow distribution with an average diameter of 9.5 and 6.6 nm, respectively. Nanoparticle size distribution shows an important effect in the electrochemical performance of the biosensor, increasing the electrochemical active surface area (EASA) and promoting the electron-transfer from the redox probe (Ferrocene/Ferrocenium) to the electrode. Furthermore, a narrow and small nanoparticle size distribution enhances the amount of antibodies immobilized on the transducer material and the performance during the detection of the PSA. Significant results were obtained for the quantification of PSA, with a limit of detection of 1 ng·ml^−1^ and sensitivities of 0.085 and 0.056 μA·mL·ng^−1^ for the two transducer materials in only 5 min of detection.

## Introduction

Prostate Specific Antigen (PSA) or Kallikrein related peptidase-3, is a serine protease secreted by the prostate gland to seminal fluid, with a single chain of 32–33 kDa. According to the World Health Organization (WHO), prostate cancer is considered the second cause of death by cancer in men, with 307,000 deaths in 2012 (Ryerson et al., 2016; Siegel et al., 2017). One of the reasons for the high mortality of this illness is the application of medical therapy in advanced stages. Normally, blood levels of this protein in healthy men is below 4 ng·mL^−1^; then, higher values in the PSA concentration are related with development of tumors in the prostate gland, considering it as a reliable biomarker for early detection of prostate cancer (Villoutreix et al., 1994; Bélanger et al., 1995; Qu et al., 2008; Liu et al., 2012).

Thus, methods for early detection are required for a proper clinical treatment. Unfortunately, current methods for cancer diagnosis, such as a histological test or screening methods based on immunoassays as an ELISA test, are time-consuming and require qualified personnel, which implies high cost (Akter et al., 2012; Wang et al., 2015). In addition, low sensibility and sensitivity makes detecting the disease difficult, especially in the first stages, where cancer shows an asymptomatic phase (Altintas et al., 2011). For that reason, many researches are trying to find low-cost techniques and improve the parameters of the detection, decreasing the time-response in the measurement.

Nowadays, sensitive detection and accurate quantification of chemical substances in physiological fluids has been a critical factor for a reliable clinical diagnostics of different diseases or medical disorders, for instance infectious diseases, diabetes melitus, Alzheimer's disease, DNA mutations, and cancer (Holliger and Hudson, 2005). Biomarkers are specific molecules (enzymes, proteins), whose concentrations increase during the development of a disease. Therefore, changes in the levels of a biomarker in physiological fluids might be related to the presence of a specific disease, and their measurement is useful for the early detection, monitoring drug therapy and medical control (Jayanthi et al., 2017).

Certainly, biosensors have been one of the most economical and functional analytical devices utilized in the last decade, as a result of their simple use in the detection of specific analytes, especially in complex samples, they are low-cost and easier handling (Turner, 2000). Biosensors are composed of two elements, a biorecognition element (enzymes, antibodies, hormones, proteins, or cells) which recognizes the target analyte, and a transducer material which converts the recognition event between the analyte and the bioreceptor in a measurable signal. Electrochemical biosensors are one of the most applied and commercialized at present (Ronkainen et al., 2010; Pisoschi, 2013).

Since the commercial implementation of electrochemical biosensors in the quantification of glucose in blood proposed by Clark and Lyons (Wang, 2002), recent advances in the biosensor field, especially in the application of nanomaterials, have brought a remarkable development in the miniaturization of electrochemical biosensors, translating in a small amount of sample required for the detection and a reduction of costs for manufacturing of the devices. Moreover, given that biological processes occur in nano and micro scales, nanostructured materials have shown an excellent platform to improve the interaction between the biological species of interest and the biosensor, guaranteeing a good measurement of the concentration. Proof of this concept has been the use of high surface area materials as metal nanoparticles (Au, Pt, Pd, Cu) (Corma and Garcia, 2008; Zhou et al., 2015), carbon materials (carbon nanotubes, graphene, and carbon nanohorns) (Wang and Dai, 2015; Bo et al., 2016), nanomanufactured electrodes, for instance interdigitated electrodes array (IDA), and screen printed electrodes (Abellán-Llobregat et al., 2017; González-Gaitán et al., 2017); producing electrochemical transducers for biosensors with high electroactive area and excellent electron transfer, key properties to provide an excellent sensitivity. At the same time, these materials might offer anchoring sites to promote the immobilization of the biorecognition element; or even promoting a direct electron transfer between the analyte and the electrode without the use of mediators or other species in the medium (Santos et al., 2015; González-Gaitán et al., 2017).

Integration between electrochemistry with other detection methods, such as enzyme immunoassays (EI), has created new platforms for biosensors with high sensibility and selectivity, benefitting from the specificity of the antigen-antibody reaction or the DNA chains hybridization, called electrochemical immunosensors, as several works have reported (Kavosi et al., 2014). Depending on the configuration of the immunosensors, they can be classified as “label-free” or “sandwich type.” The first ones employ the interaction of the antibody (Ab) as biorecognition element with the antigen (Ag) to create an immunocomplex (Ab-Ag) onto the surface which will block the surface for electron transfer of some electroactive species (Okuno et al., 2007; Wang et al., 2013). On the other hand, sandwich type uses the coupling of a second antibody with antigen immobilized, creating an immunocomplex Ab_1_-Ag-Ab_2_, to increase the electrical barrier of the system. However, several works have developed sandwich type immunosensors with a second antibody labeled with peroxidase or phosphatase enzymes which catalyzes the hydrogen peroxide reduction, creating an increase in the electrochemical signal (Yu et al., 2006); (Yan et al., 2012).

Even though PSA detection has been widely studied using different electrochemical techniques, the high time consumption to quantify the concentration of the analyte has not been solved, taking at least 24 h for its analysis.

In this work a label-free electrochemical platform has been studied for the fast measurement of concentrations of PSA for the identification of the biomarker. Then, electrochemical detection of PSA was carried out with glassy carbon electrodes (GC) modified with functionalized multi-wall carbon nanotubes decorated with gold nanoparticles and the immobilization of monoclonal antibodies to the PSA.

## Experimental Section

### Reagents and Equipment

Multi-Wall Carbon Nanotubes (MWCNT) with purity 95% (8 nm of diameter) and 10–30 μm length were purchased to Cheap Tubes Inc. (Cambridgeport, USA). Nitric acid (65%) from Panreac was employed to functionalize and purify the carbon nanotubes. Purified mouse monoclonal PSA antibody (Ab) (Purified IgG-Ab) and native human PSA purified were purchased from Bio-Rad Laboratories (Munich, Germany).

Potassium dihydrogen phosphate (KH_2_PO_4_) and dipotassium hydrogen phosphate (K_2_HPO_4_) obtained from Merck and VWR Chemicals, respectively, were used to prepare phosphate buffer solutions (0.01 M PBS, pH = 7.2 and 0.1 M PBS, pH = 7.2) to dissolve the immunoreagents and as electrolyte, unless otherwise noted. Ferrocenium hexafluorophosphate (Fc-97%), employed as redox probe was purchased from Sigma Aldrich. All the solutions were prepared using ultrapure water (18 MOhm·cm, Purelab Ultra Elga equipment). The gases N_2_ (99.999%) and H_2_ (99.999%) were provided by Air Liquide.

Reagents employed in the gold nanoparticles synthesis included sodium tetrachloroaurate (III) dihydrate *(*NaAuCl_4_·2H_2_O, 99%*)*, poly-n-vinylpyrrolidone (PVP, 40K), sodium hydroxide (NaOH, 99,99% purity), anhydrous ethylene glycol and methanol (+98%) and were purchased from Sigma-Aldrich.

### Preparation of the Transducer Material. Functionalized Multiwall Carbon Nanotubes (fMWCNT) With Gold Nanoparticles (AuNPs)

#### Functionalization of Multi-Wall Carbon Nanotubes

Multi-Wall carbon nanotubes (MWCNT) were subjected to a functionalization treatment by oxidation in nitric acid solution, according to the following procedure. In a two-necked, round-bottom flask, 200 mg of MWCNT were added in 100 mL of 3 M HNO_3_ at 120°C for 24 h under reflux conditions.

MWCNTs were extracted after 24 h, filtered, washed with ultrapure water until the pH was neutral and dried in vacuum at 60°C for 24 h, and weighed. The sample was called fMWCNTs. These fMWCNTs were dispersed in water using sonication bath for 10 min, to get a concentration of 1 mg·mL^−1^.

#### Gold Nanoparticles Synthesis

Gold nanoparticles were synthesized following the reduction-by-solvent method (Lu et al., 1999), adapting a previously published procedure (Domínguez-Domínguez et al., 2006). Given that, this procedure allows a control of the nanoparticle size distribution varying the molar ratio PVP/Au. In this research, two syntheses were carried out using two PVP/Au molar ratios (0.5 and 50). At the same time, all the chemical reactions during the synthesis were carried out in an inert atmosphere of argon, using a Schlenk system to avoid undesirable reactions. A typical synthesis is carried out as described in section S1.1 in supporting information (see Figure S1).

#### fMWCNT Decorated With AuNPs Dispersion

Transducer materials were prepared by the impregnation method in liquid phase, where the suspensions of the carbon material were put in contact with the nanoparticles colloid. Suspensions of fMWCNT (1 mg·mL^−1^) were mixed with an appropriate amount of purified gold nanoparticles suspension (1 mg·mL^−1^) to yield 5% (w/w) of metal loading. The dispersions were sonicated and stirred overnight in order to ensure the adsorption of the metal nanoparticles. Samples were filtered in vacuum to remove non-adsorbed nanoparticles and dried in vacuum at 60°C for 24 h. Based on the ratio PVP/Au used in the synthesis, transducer material will be named as fMWCNT-AuNPs-0.5 ratio and fMWCNT-AuNPs-50 ratio, for 0.5 and 50 ratios, respectively.

### Immunosensor Electrode Preparation

**Scheme 1:**
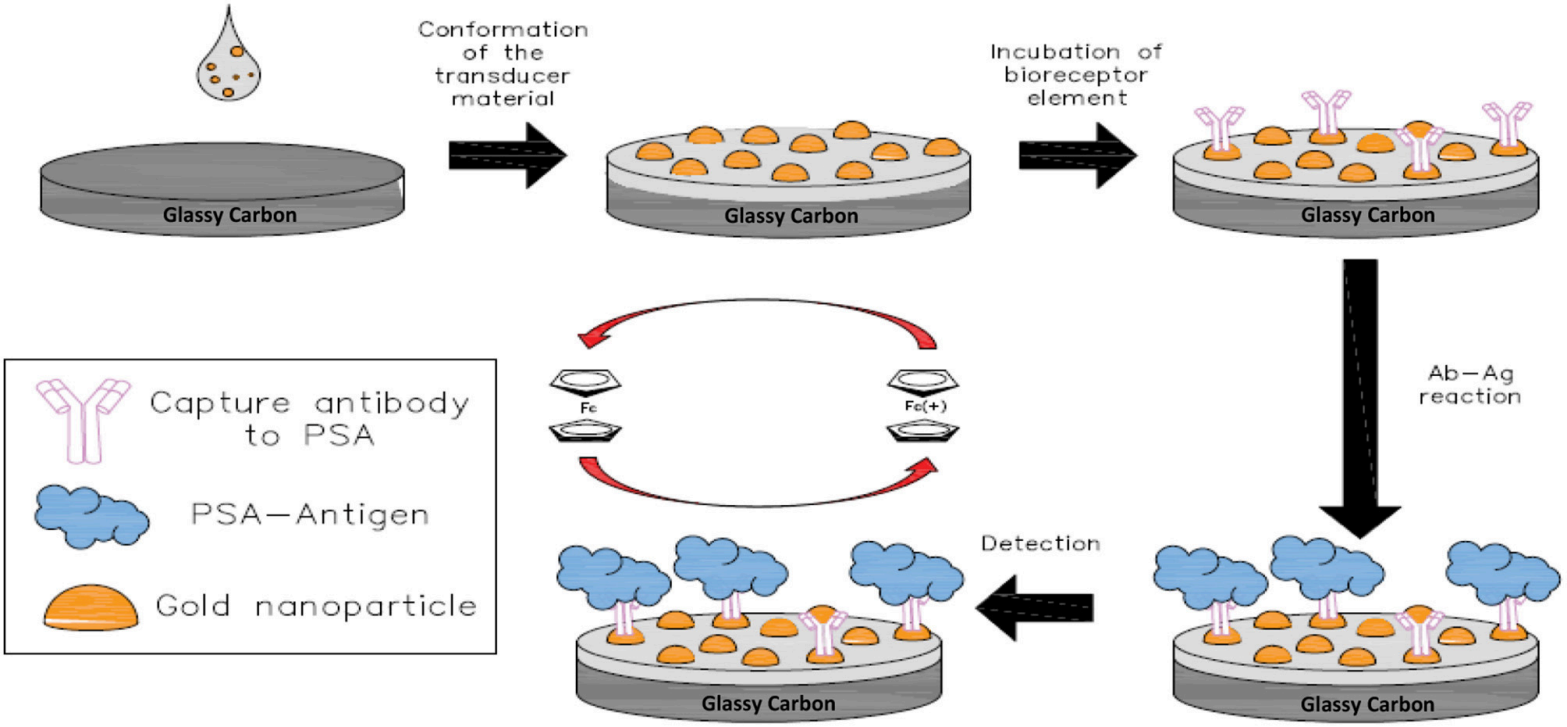
Illustration of the stepwise process for PSA immunosensor electrode fabrication and detection of the cancer biomarker.

A schematic diagram of the stepwise assembly procedure of the immunosensor is shown in Scheme 1. Prior to the modification, glassy carbon electrodes surface (3 mm diameter) was sanded with emery paper and polished using 1 and 0.05 μm alumina slurries, then rinsed with ultrapure water. Ten milligrams of the transducer material (fMWCNT-AuNPs) were dispersed in water with the aid of ultrasonic bath for 45 min, using an ice bath to avoid heating during the sonication. A 4 μL aliquot of the dispersion was dropped onto the glassy carbon (GC) surface and dried under an infrared lamp to remove the water. This procedure was repeated 3 times until completing 12 μL of the carbon material suspension on the electrode. Then, 5 μL of monoclonal antibodies solution (10 μg·mL^−1^) were added onto the electrode surface and incubated at 4°C for 24 h, yielding the GC-fMWCNT-AuNPs-Ab electrode with 4.16 μg Ab·${\text{g}}_{\text{fMWCNT}}^{-1}$ of loading. Subsequently, electrodes were rinsed with PBS (0.01 M, pH = 7.2) to remove all non-reacted material. Afterwards, the electrodes were stored in PBS (0.1M, pH = 7.2) solution at 4°C before electrochemical detection of PSA in 0.1 M PBS + 0.5 mM Fc (pH = 7.2) by chronoamperometry.

### Electrochemical Methods

Electrochemical characterization was performed in an EG&G Princeton Applied Research Model 263A Potentiostat/Galvonastat using a standard three-electrode cell configuration, in which GC-fMWCNT-AuNPs-Ab electrode was the working electrode (WE), a gold wire as counter electrode (CE), and a reversible hydrogen electrode (RHE) introduced in the same electrolyte as reference electrode (RE). All the measurements were carried out in 0.1 M PBS (pH = 7.2) and 0.1 M PBS + 0.5 mM Fc (pH = 7.2) solutions, deoxygenating the cell during the measurement by bubbling nitrogen. Previously, fMWCNT-AuNPs were submitted to a continuous cycling in 0.1 M PBS (pH = 7.2) to clean the electrode.

The electrochemical detection of PSA was carried out by chronoamperometry in a BIOLOGIC SP-300 potentiostat, applying a steady potential of 1.0 V in 0.1 M PBS + 0.5 mM Fc (pH = 7.2) solution. A total of 8–9 aliquots of PSA solution (500 ng·mL^−1^) were added to the electrochemical cell, achieving concentrations between 1 and 10 ng·mL^−1^. Three minutes of reaction were maintained after the addition of each aliquot under stirring during the immunoreaction to ensure a good homogenization of the analyte in the electrolyte and promoting the transport of the PSA to the electrode.

All the calibration curves and the electrochemical characterization, including the immobilization process, were performed by triplicate using 3 different electrodes, synthesized separately. Error bars are incorporated in the calibration curves considering the standard deviation. Afterwards the electrochemical determination of PSA, mass of carbon nanotubes modified with AuNPs were determined using the gravimetric capacitance in PBS; in this way, current was normalized to the mass to avoid effect of mass.

### Physicochemical Characterization

Transmission electron microscopic measurements (TEM) were carried out using JEOL TEM, JEM-2010 model, which is equipped with and Oxford X-ray detector (EDS), INCA Energy TEM 100 model, and GATAN acquisition camera.

X-Ray photoelectron spectroscopy (XPS) was performed in a VG-Microtech Mutilab 3,000 spectrometer and Al Kα radiation (1253.6 eV). The deconvolution of the XPS Au4f, C1s, S2p, and N1s was done by least squares fitting using Gaussian-Lorentzian curves, while a Shirley line was used for the background determination. The S2p spectra have been analyzed considering the spin-orbit splitting into S2p3/2 and S2p1/2 with a 2:1 peak area ratio and 1.2 eV splitting (Castner et al., 1996). The XPS measurements were done in different parts of a given sample and repeated in two different samples, being the results similar.

To determine metal content, 10 mg of the carbon material modified with AuNPs were digested in an acid solution [1 HNO_3_ (65%):3 HCl (37%)]. The suspension was sonicated for 20 min and heated at 80°C for 6 h until evaporation. Afterwards, 2 mL of HNO_3_ were added and diluted with ultrapure water. Solutions were then analyzed using inductively coupled plasma optical emission spectroscopy (ICP-OES), Perkin-Elmer Optima 4,300.

## Results And Discussion

### fMWCNT-AuNPs Electrodes Characterization

#### Physicochemical Characterization

MWCNT pristine material and fMWCNT were studied by temperature programmed desorption (TPD) to observe the nature of the different oxygen surface groups incorporated during the functionalization treatment and by Field Emission Scanning Electron Microscopy (FE-SEM) for studying possible morphological changes in the structure of the carbon material. The most relevant results are presented in section S2.1 in supporting information (see Figures S2–S4 and S6, Table S1 and discussion included in supporting information).

**Figure 1:**
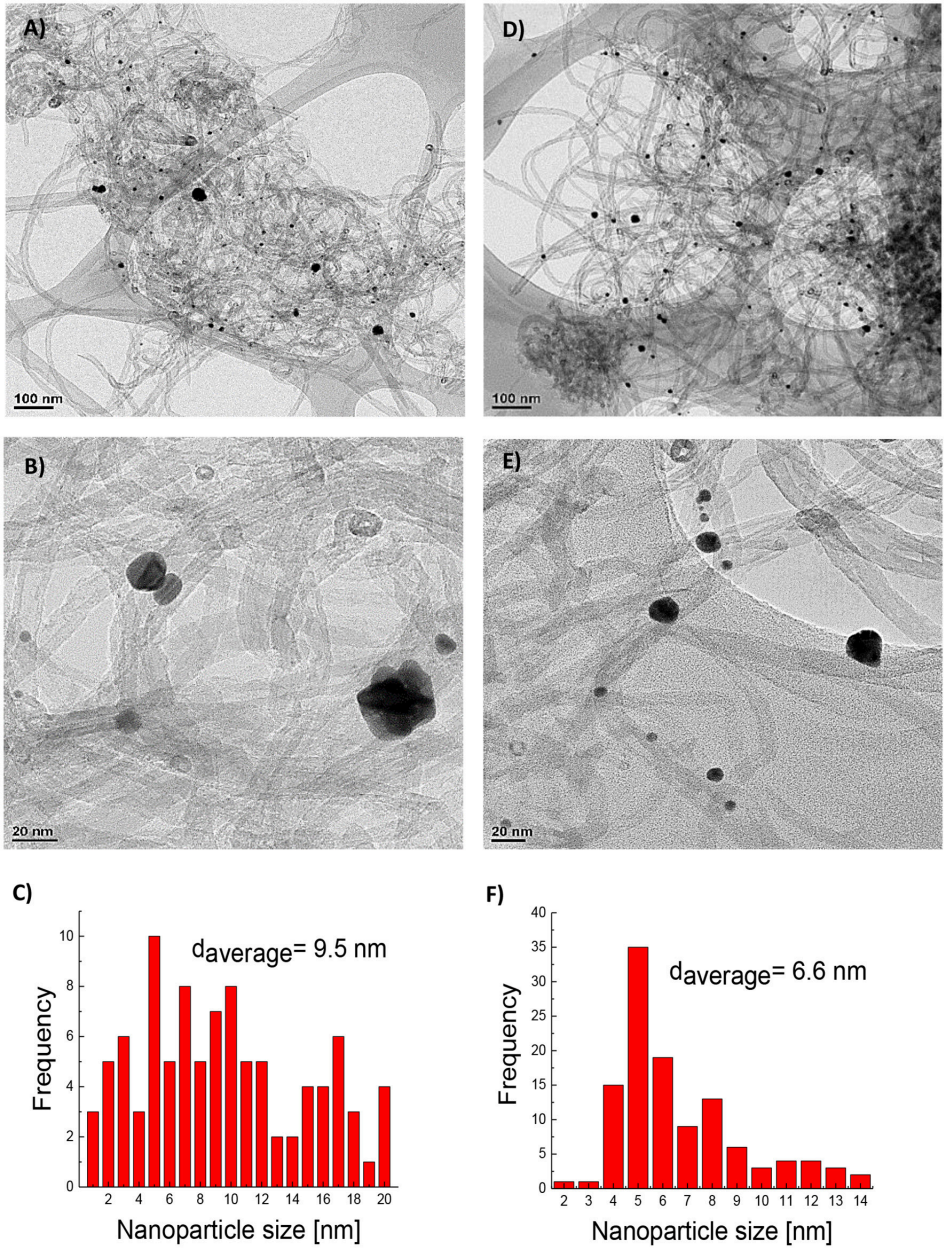
**(A–F)** HR-TEM micrographs: **(A)** fMWCNT-AuNPs-0.5, **(B)** fMWCNT-AuNPs-0.5 magnified, **(C)** histogram of Au nanoparticle size distribution in fMWCNT-AuNPs-0.5 transducer material, **(D)** fMWCNT-AuNPs-50, **(E)** fMWCNT-AuNPs-50 magnified, and **(F)** histogram of Au nanoparticle size distribution in fMWCNT-AuNPs-50 transducer material.

Figure 1 shows the TEM micrographs of the carbon materials with AuNPs. This Figure reveals the distribution and particle size of the AuNPs onto the surface of the carbon nanotubes after the impregnation procedure. As previously reported, PVP concentration during the synthesis of the metal nanoparticles is a key factor to control the nanoparticle size in the colloid (Bönnemann and Richards, 2001; Miguel-García et al., 2010). Figures 1C,F show the particle size distribution determined by TEM. As expected, the nanoparticle size distribution decreases to a narrow distribution with the increase of the amount of PVP. The average particle size changes from 9.5 to 6.6 nm with the increase in the PVP/Au ratio. Moreover, agglomeration and a non-spherical shape of the nanoparticles is observed for lower PVP/Au ratio.

Gold loading was quantified by ICP-OES, achieving values of 2.1 and 3.6 wt % for the ratios PVP/Au of 0.5 and 50, respectively (See Table 1). Furthermore, XPS spectra for Au-4f core level region of our samples in Figure S5, shows two doublets at 84.1 and 87.8 eV associated with Au^0^ species and at 84.9 and 88.6 eV related with a higher oxidized state (Au^+δ^ species) (Jasmin et al., 2017; Liberman et al., 2017). The Au^0^/Au^+δ^ ratio is for both samples 90.5%.

**Table 1:** Amount of Au obtained by ICP and EASA in the fMWCNT-AuNPs and fMWCNT-AuNPs-Ab materials.

**Sample**	**Au ICP-OES (wt %)**	**EASA (m^**2**^ g^**−1**^)**	**S-2p amount by XPS (wt %)**	**% of Au-S species in the total S-2p**
fMWCNT-AuNP-0.5	2.1	28.6	–	–
fMWCNT-AuNP-50	3.6	33.4	–	–
fMWCNT-AuNP-0.5-Ab	–	–	0.16	9
fMWCNT-AuNP-50-Ab	–	–	0.32	28

*Amount of S obtained by XPS and percentage of thiol group bound to Au*.

#### Electrochemical Characterization of fMWCNT-AuNPs

**Figure 2:**
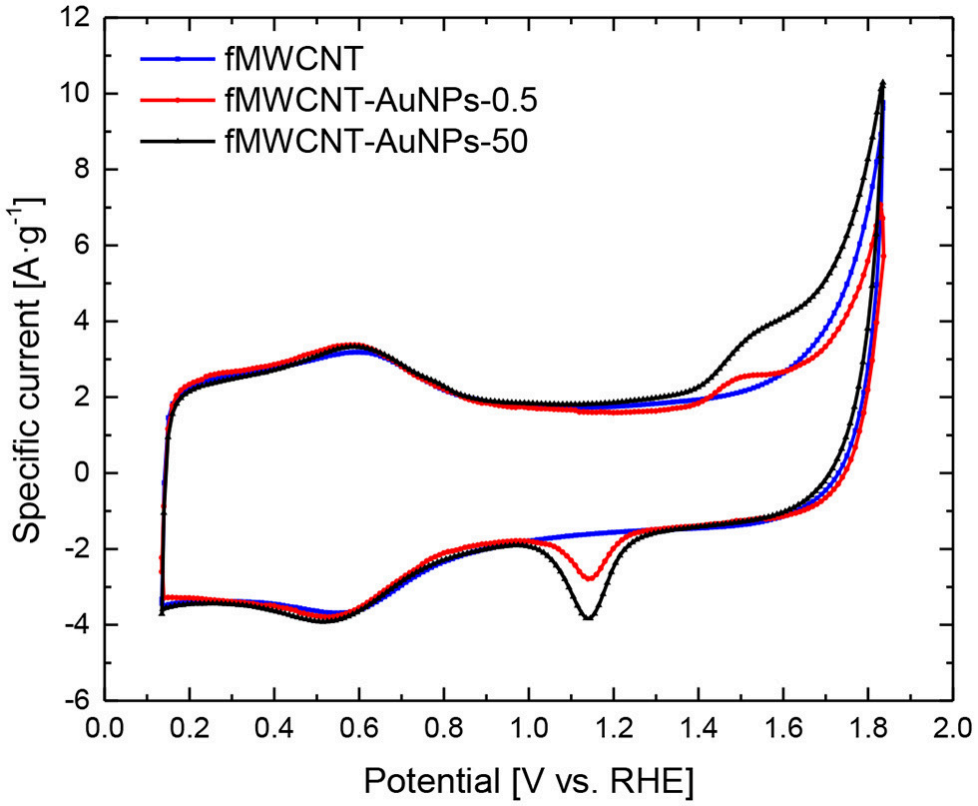
CV for fMWCNT, fMWCNT-AuNPs-0.5 and fMWCNT-AuNPs-50 in 0.1 M PBS (pH = 7.2) and v_scan_ = 50 mV·s^−1^.

Figure 2 shows the cyclic voltammograms (CV) for fMWCNT and fMWCNT-AuNPs modified GC electrode between 0.1 and 1.8 V. The CV for fMWCNT shows an oxidation peak at ~ 0.6 V, with the corresponding reduction peak at 0.55 V; which are associated with the surface oxygen groups formed during the functionalization treatment. At the same time, the capacitance of these fMWCNTs is 43 F·g^−1^, a value similar to that previously reported in similar conditions (González-Gaitán et al., 2017). However, incorporation of the gold nanoparticles in the carbon material generate an additional oxidation process at 1.5 V and the corresponding reduction at 1.15 V, corresponding with the oxidation-reduction of the gold oxide in the surface of the nanoparticles (Sukeri et al., 2015).

It is well-known that nanoparticle size has an important influence in the electrochemical active surface area (EASA) and smaller nanoparticle size implies an increase in the EASA (Zaragoza-Martín et al., 2007; Sukeri et al., 2015; Ayán-Varela et al., 2017). Table 1 shows the EASA for the different transducer material synthesized in this work. It can be observed that the decrease in the particle size implies an increase in the EASA of around 17%.

### Electrochemical Characterization of the Monoclonal Antibodies Immobilized on the fMWCNT-AuNPs Samples

**Figure 3:**
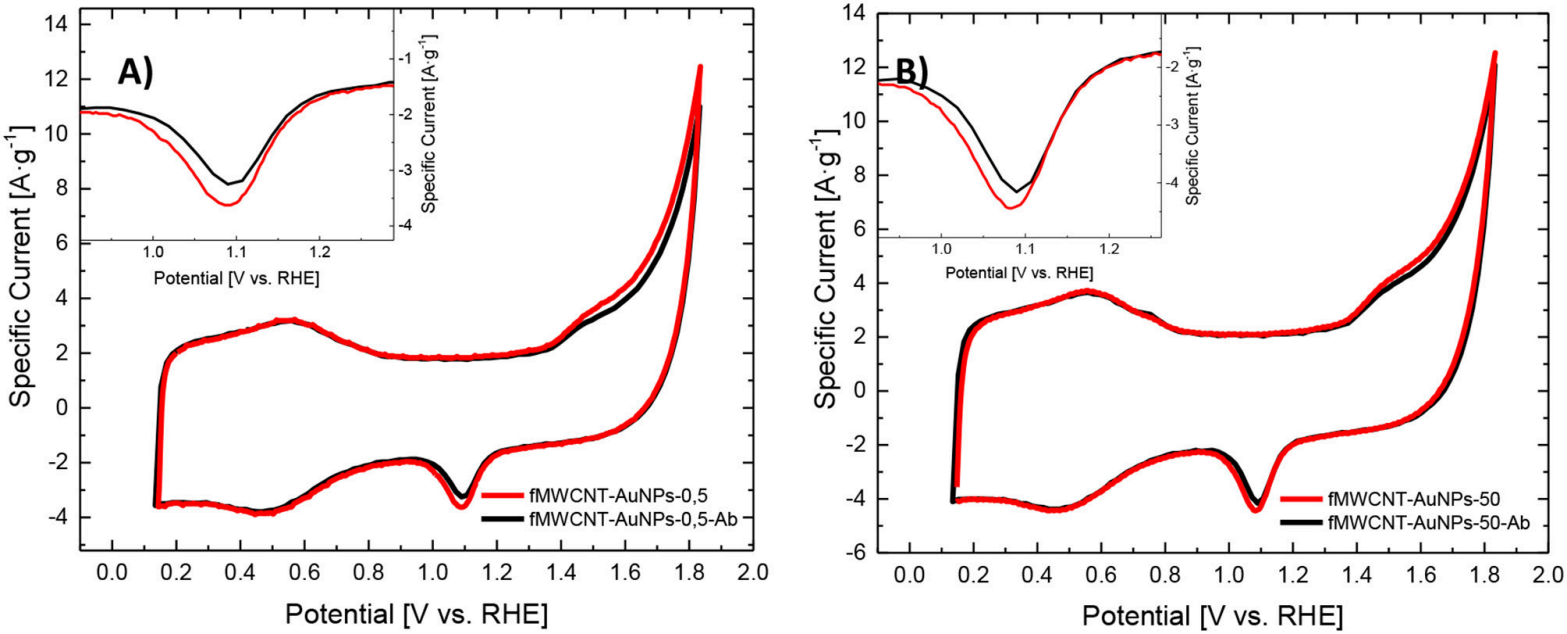
**(A,B)** Electrochemical behavior of the immunosensor fMWCNT-AuNPs-Ab: **(A)** CV for transducer material before (red line) and after (black line) the immobilization with monoclonal antibodies of fMWCNT-AuNPs-0.5 in 0.1 M PBS (pH = 7.2), v_scan_ = 50 mV·s^−1^, **(B)** CV for transducer material before (red line) and after (black line) the immobilization with monoclonal antibodies of fMWCNT-AuNPs-50 in 0.1 M PBS (pH = 7.2), v_scan_ = 50 mV·s^−1^.

Figure 3 shows the CV for the fMWCNT-AuNP electrode before and after immobilization of antibodies. It can be observed that no significant changes are appreciated on the double layer region of carbon nanotubes between 0.1 and 0.75 V for both materials. This result suggests that the immobilization of the antibody is not produced on the carbon nanotube surface. On the other hand, the redox processes for gold surface oxide shows a decrease in the current indicating a blockage of the surface area of AuNP and a decrease in the EASA of 16 and 24% for fMWCNT-AuNPs-0.5 (Figure 3A) and fMWCNT-AuNPs-50 (Figure 3B), respectively (Dey et al., 2012; Deiminiat et al., 2017). Section S2.2 of the supporting information includes, for comparison purposes, the electrochemical behavior of a polycrystalline gold electrode modified with monoclonal antibodies to PSA (see Figures S7 and S8). The high affinity between thiol groups present in the antibody structure with the gold nanoparticles causes the immobilization of the antibody (Benvidi et al., 2015) producing the observed decrease in available Au surface area.

**Figure 4:**
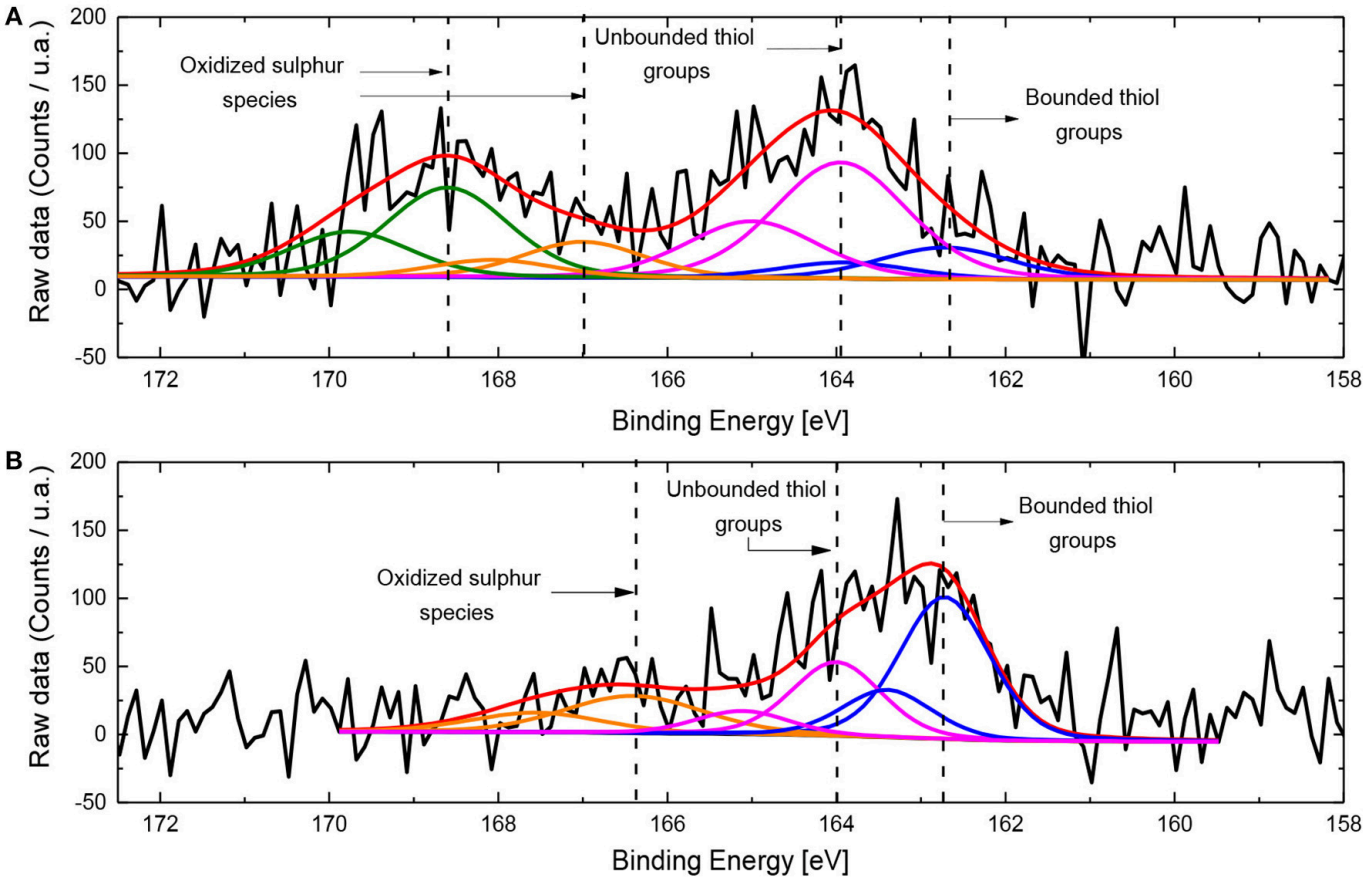
**(A,B)** XPS spectra for S2p: **(A)** fMWCNT-AuNPs-0.5 and **(B)** fMWCNT-AuNPs-50 synthesized.

The covalent interaction between gold and the bioreceptor can be observed in the S2p spectra after immobilization (Figure 4), where, S2p3/2 peaks at binding energies of 163.9 and 162.7 eV, can be associated unbound and bound thiol groups (Castner et al., 1996). Considering the low amount of sulfur species, the XPS has significant noise and the deconvolution can only be considered as qualitative. The bound thiol species can be associated to the interaction between the antibody and the gold surface (Yam et al., 2003; Berner et al., 2007; Amendola et al., 2014; Venditti et al., 2017). Then, it can be suggested that the interaction between gold nanoparticles and thiol groups promote the covalent immobilization of the antibodies in the material. The samples with PVP/ratio of 50 presents a much higher intensity of the peak at 162.7 eV, indicating a higher quantity of thiol groups bonded to AuNPs, in contrast with the AuNPs prepared with a lower PVP/Au ratio. This suggests that a higher amount of antibodies are immobilized on the surface of the electrode which contains the smaller average Au nanoparticle size (Table 1). Moreover, the presence of a higher amount of covalently bounded Ab-Au in the fMWCNT-AuNPs-50-Ab samples permits either a proper immobilization or even a better orientation of the bioreceptor in the transducer (Trilling et al., 2013). Even though the interaction with thiol generates changes in the oxidation states of gold from Au^0^ to Au^+δ^, authors have observed that the signal of Au^0^ can scatter the intensity of the Au^+δ^, when low concentration of this latter species is present. For that reason no significant changes in gold spectra can be observed (Singh and Whitten, 2008).

Additional signals in the S2p spectra appear at higher binding energies, especially for fMWCNT-AuNPs-0.5-Ab, between 166.4 and 169.6 eV, which can be related with oxidized sulfur species, probably associated with the denaturalization which takes place in the antibody or other direct interactions with the functional groups of the carbon material. These interactions are more probable for the sample with the largest AuNPs size and this produces a larger proportion of inappropriate immobilization of the biorecognition element in the surface of the material (Singh and Whitten, 2008; Trilling et al., 2013).

#### Electrochemical Performance of the PSA Immunosensor

Despite the blocking effect of the Ab antibodies in the AuNPs surface and subsequent formation of the immunocomplex antibody-antigen, which can be used as a label-free platform for detection, application of the high potential to achieve the oxidation-reduction reaction of gold nanoparticles produces the denaturalization and desorption of the bioreceptor [See Preparation of the transducer material. Functionalized multiwall carbon nanotubes (fMWCNT) with gold nanoparticles (AuNPs)]. Then, a redox mediator could be used in order to decrease the detection potential (Chuah et al., 2012), for this part ferrocene was used as redox mediator for the sensing process of the biomarker.

**Figure 5:**
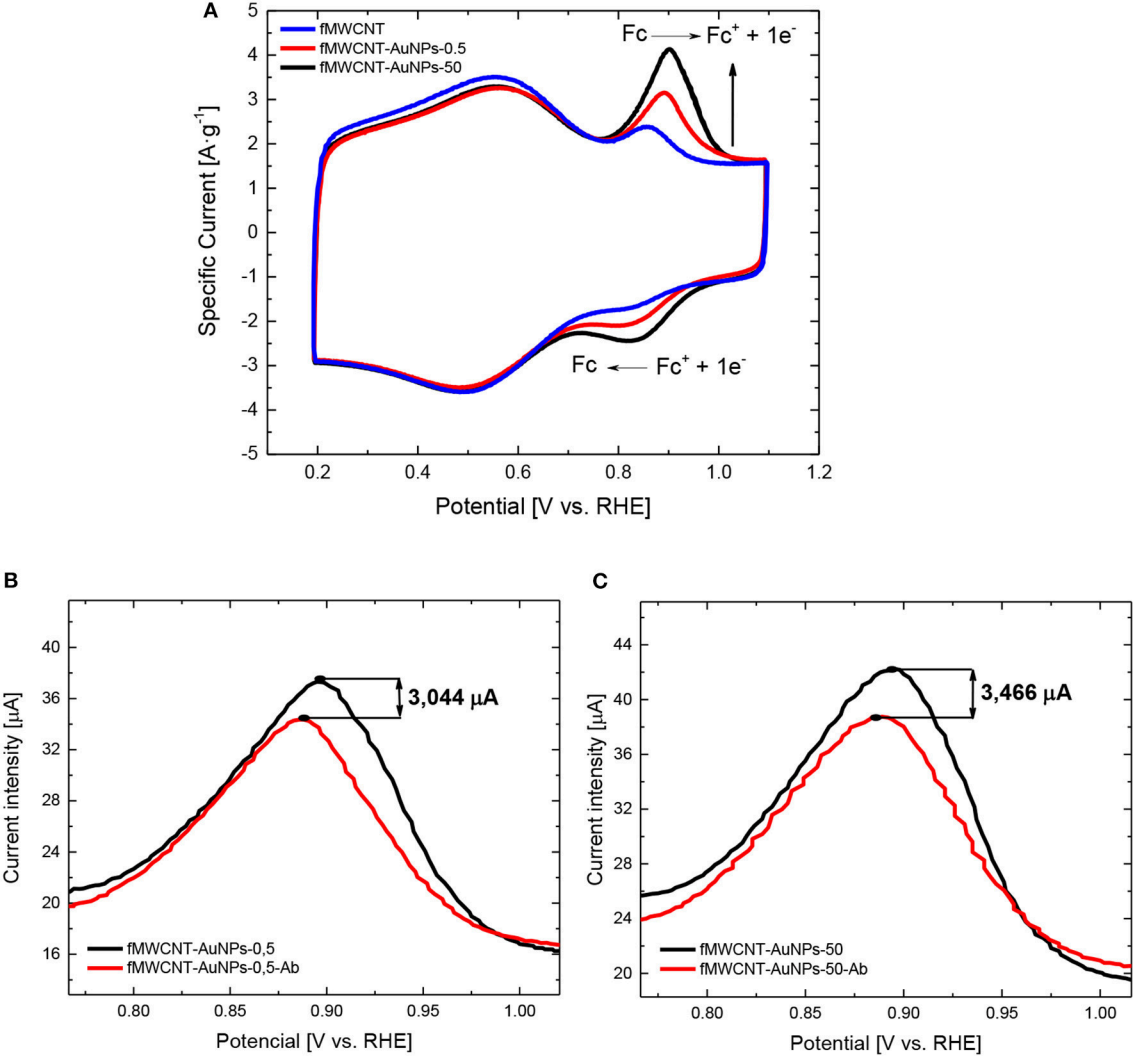
**(A–C)** Electrochemical behavior of material synthesized in ferrocene: **(A)** CV for fMWCNT and fMWNCT-AuNP in PBS (0.1 M, 0.5 mM Fc, pH = 7.2) and v_scan_ = 50 mV·s^−1^, **(B)** CV for transducer material in the oxidation peak before (red line) and after (black line) the immobilization with monoclonal antibodies of fMWCNT-AuNPs-0.5 in 0.1 M PBS + 0.5 mM Fc (pH = 7.2) and v_scan_ = 50 mV/s, **(C)** CV for transducer material in the oxidation peak before (red line) and after (black line) the immobilization with monoclonal antibodies of fMWCNT-AuNPs-50 in 0.1 M PBS + 0.5 mM Fc (pH = 7.2) and v_scan_ = 50 mV/s.

Figure 5A shows the CVs for the fMWCNT and fMWCNT-AuNPs with different PVP/Au ratios (0.5 and 50) in 0.1 M PBS + 0.5 mM Fc solution, where the corresponding oxidation-reduction processes of ferrocene at 0.9 and 0.83 V can be observed. Current density for both processes increases with the incorporation of the AuNPs. Moreover, increasing the PVP/Au ratio causes a growth in the current density for both processes, as a result of a larger EASA.

As reported, the oxidation process of ferrocene in aqueous solution is characterized by a single-electron transfer mechanism preceded by a weak adsorption process (Salinas-Torres et al., 2011). In this case, the separation peak for the redox process of ferrocene in fMWCNT at 50 mV·s^−1^ is around 47 ± 1 mV, suggesting that the mechanism for the redox process of ferrocene involves adsorption on the carbon material. On the contrary, incorporation of the AuNPs in the carbon material causes an increase of the separation peak to around 72 ± 1 mV, suggesting that the catalyst avoids the adsorption step during the oxidation process, making the process more irreversible (Sieben et al., 2014). The electrochemical behavior with the scan rate for the different transducer material synthesized in presence of the Fc can be observed in section S2.3 of the Supporting Information (see Figures S9 and S10).

Figures 5B, C show the decrease in the peak current for the oxidation process at 0.9 V for electrodes modified with Ab monoclonal antibodies, suggesting that the Fc redox processes are electrochemically impeded, presenting the higher current drop in fMWCNT-AuNPs-50-Ab biosensors. Some authors have suggested that, steric effects of the different functional groups of the antibodies immobilized on the surface, reduce the electron transfer between the electrode and the electroactive species (Fc), which might be used as a label-free platform for detection of the biomarker. These results agree with the XPS results and confirm the presence of the Ab on the surface of AuNPs (Deiminiat et al., 2017).

**Figure 6:**
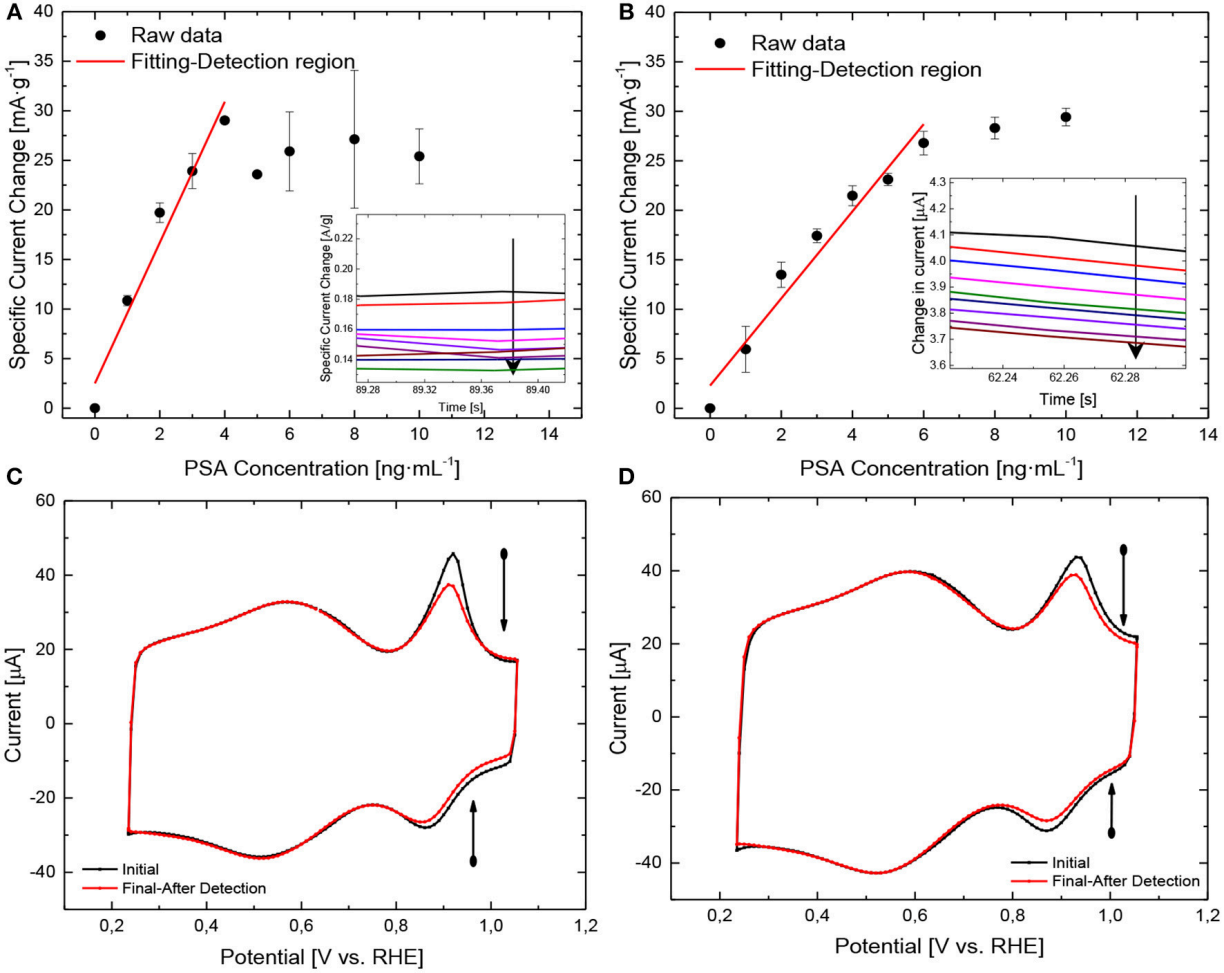
(**A–D)** Electrochemical detection of the biomarker: **(A)** Calibration curve for fMWNCT-AuNPs-0.5-Ab, **(B)** Calibration curve for fMWNCT-AuNPs-50-Ab. All the measurements were performed in 0.1 M PBS + 0.5 mM Fc (pH = 7.2) and v_scan_ = 50 mV·s^−1^, **(C)** CV for fMWNCT-AuNPs-0.5-Ab before (black line) and after (red line) detection of PSA in 0.1 M PBS + 0.5 mM Fc (pH = 7.2) and v_scan_ = 50 mV/s, **(D)** CV for fMWNCT-AuNPs-50-Ab before (black line) and after (red line) detection of PSA in 0.1 M PBS + 0.5 mM Fc (pH = 7.2) and v_scan_ = 50 mV/s.

Immunosensor performance was investigated using chronoamperometry at 1 V with different concentrations of PSA in 0.1 M PBS + 0.5 mM Fc solution. Only 3 min were left between the additions of each aliquot for reaction between Ab immobilized in the AuNPs and PSA in solution. A decrease of the oxidation current associated to Fc is obtained with the increase of the PSA concentration. The change of the current with the addition of PSA can be used as electrochemical signal for detection of PSA. Given that antibody-antigen (Ab-Ag) reaction takes place after the addition of PSA in the solution, the immunocomplex Ab-Ag on the surface of the biosensor might increase the steric effects onto the surface of the electrode, which is translated in a decrease of the current for the ferrocene oxidation process (Fc → Fc + 1e^−^) (Torati et al., 2017). The calibration curves for both electrodes are shown in Figures 6A,B, with the chronoamperometric profiles (inset). First of all, decrease in the electrical current density of the oxidation process for ferrocene is observed with the increase of the concentration of PSA, demonstrating the blocking of the surface by the Ab-Ag complex which was mentioned above. Furthermore, all immunosensors show a typical Langmuir behavior, where an initial linear range can be observed and a plateau where the saturation of the biorecognition element takes place. In this case, saturation occurs as a consequence of the interaction of most of the immobilized antibodies with the antigen in solution, therefore, no more active sites are available for an enhanced detection of PSA after achieving this saturation and thus no current changes happen. Finally, only an important decrease in the oxidation-reduction processes for ferrocene can be observed in the cyclic voltammograms Figures 6C, D, before and after the sensing process, as result of the immune-complex onto the surface which blocks the electron transfer, proving the biosensing activity of the immunosensor synthetized.

The saturation range of the immunosensor takes place at higher concentration values with the increase of PVP/Au ratio, showing at the same time, a higher analytical linear range detection of (0–4 ng·mL^−1^) for fMWNCT-AuNPs-0.5-Ab and (0–6 ng·mL^−1^) for fMWCNT-AuNPs-50-Ab. This behavior can be attributed to the nanoparticle size distribution in the fMWCNT-AuNPs-50-Ab immunosensor, which can provide a higher surface area, promoting a higher amount of biorecognition element immobilized onto the surface, a critical factor in the proper interaction of the fragment antigen-binding (Fab) part of the antibody with the antigen, which is in concordance with the XPS spectra for sulfur observed in section Electrochemical characterization of the monoclonal antibodies immobilized on the fMWCNT-AuNPs samples (Tajima et al., 2011). The linear regression of the calibration curve for both electrodes (fMWCNT-AuNPs-0.5-Ab and fMWCNT-AuNPs-50-Ab) were: Δi (mA/g _fMWCNT_) = 7.112 C_PSA_ (ng _PSA_·ml^−1^) + 2.478 and Δi (mA/g _fMWCNT_) = 4.743 C_PSA_ (ng _PSA_·ml^−1^) + 1.717, respectively and with the same correlation coefficient (*R*^2^ = 0.96) for both electrodes. On the other hand, the sensitivity of the immunosensor shows a higher value in fMWNCT-AuNPs-0.5-Ab biosensors, in comparison with the samples with the highest PVP/Au ratio, which can be attributed to the fast decrease in the amount of antibodies in the electrode during the detection process, which is also supported by the saturation limit of the immunosensor.

Table 2 shows the analytical parameters obtained with both immunosensors. It can be observed that the sensitivity is higher for the fMWCNT-AuNPs-0.5 electrodes; however, the linear range is higher for the fMWCNT-Au-50 electrode.

**Table 2:** Analytical figures of merit for the quantification of PSA with both electrochemical modified electrodes (fMWCNT-AuNPs-0.5-Ab and fMWCNT-AuNPs-50-Ab).

**Parameter**	**Sample**	
	**fMWCNT-AuNP-0.5-Ab**	**fMWCNT-AuNP-50-Ab**
Sensitivity [(mA·g_fMWCNT_^−1^)/(ng·mL^−1^)]	7.11 ± 0.82	4.74 ± 0.43
Intercept (ng·mL^−1^)	2.48 ± 2.02	2.32 ± 1.22
R	0.96	0.96
N	5	7
Linear range (ng·mL^−1^)	0–4	0–6
LOD (ng·mL^−1^)	1	1
LOQ (ng·mL^−1^)	3.3	3.3

Table 3 shows a comparison of some analytical parameters obtained in this work, with data published in the literature. In most of the cases, the sensitivity is similar or higher than the published, being the proposed sensor competitive with respect to previously reported electrochemical sensors. Moreover, time consumption to detect the PSA is greatly improved with respect to other immunosensors.

**Table 3:** Analytical parameters for different PSA biosensors.

**Electrode**	**Sample**				
	**Electrochemical technique (Time to measure)**	**Detection limit (ng·mL^**−1**^)**	**Concentration range (ng·mL^**−1**^)**	**Sensitivity (μA·mL·ng^**−1**^)**	**References**
fMWCNT-AuNPs-0.5-Ab	Amperometric (5 min)	1	0–4	0.085	This work
fMWCNT-AuNPs-50-Ab	Amperometric (5 min)	1	0–6	0.056	This work
GC-MWCNT-Reactor	Amperometric (24 h)	1	0–60	0.047	Panini et al., 2008
AuNPs-PANAM dendrimer/MWCNTs/Chitosan/Ionic liquid	Voltametric (24 h)	0.05	0.05–2 (Range 1)	0.0942	(Kavosi et al., 2014)
Pt/Ti patterned-SWCNT	DPV (24 h)	0.25	0–1	…	Okuno et al., 2007

## Conclusions

Electrochemical PSA sensors based on fMWCNT-AuNPs-Ab with different nanoparticle size distribution were developed. In order to study the effect of the nanoparticle size in the performance of the electrochemical sensor, two different synthesis were carried out, controlling the PVP/Au molar ratio to obtain gold nanoparticles with a wide and narrow distribution, and an average diameter of 9.5 nm and 6.6 nm for PVP/Au ratios of 0.5 and 50, respectively. Incorporation of the metal nanoparticles was verified by CVs, which demonstrated that narrow nanoparticle size distribution has higher EASA.

The prepared electrodes with different Au nanoparticles sizes showed a decrease of the current density of the redox processes associated with the gold oxide formation after the immobilization process of the biorecognition element, as a consequence of the blocking effect of this molecule in the surface. This behavior was shown for ferrocene (Fc-Fc^+^) employed as redox probe. At the same time, XPS spectra for S2p demonstrated the presence of the S-Au bound, due to the covalent immobilization of the antibodies on the nanoparticle surface. Moreover, narrow Au nanoparticle size distribution promotes a higher immobilization of the antibodies which was seen as an increase in the amount of S-Au bound.

In this work, the electrochemical detection of PSA by chronoamperometry provided a fast method for the detection of this compound, which profits from the steric effects in the surface of the electrode created by the formation of the immunocomplex antibody-antigen, which acts as a diffusional barrier for the electroactive species, which is translated in a decrease in the current intensity of the oxidation processes. Even though a decrease in current with the increase of PSA concentration can be observed in both biosensors, the lineal range and saturation concentrations is influenced by the nanoparticle size, being fMWCNT-AuNPs-50-Ab the sample which presents the best performance with a higher linear range between 0 and 6 ng·mL^−1^ with a good sensitivity of 4.74 mA g^−1^/ng mL^−1^, allowing the detection in human samples.

## Author Contributions

All authors listed have made a substantial, direct and intellectual contribution to the work, and approved it for publication.

### Conflict of Interest Statement

The authors declare that the research was conducted in the absence of any commercial or financial relationships that could be construed as a potential conflict of interest.
