# Supplementary material for: Carbon Nanotubes Modified With Au for Electrochemical Detection of Prostate Specific Antigen: Effect of Au Nanoparticle Size Distribution
Source: Front Chem. 2019 Mar 27;7:147. doi: 10.3389/fchem.2019.00147 (PMC6445958; doi:10.3389/fchem.2019.00147)
Supplement: Supplementary file 1 [file Table_1.DOCX]

**CARBON NANOTUBES MODIFIED WITH Au FOR ELECTROCHEMICAL DETECTION OF PROSTATE SPECIFIC ANTIGEN: EFFECT OF Au NANOPARTICLE SIZE DISTRIBUTION**

Andrés Felipe Quintero-Jaime^a^, A. Berenguer-Murcia^b^, Diego Cazorla-Amorós^b^, Emilia Morallón^a^.

^a^ Departamento de Química Física and Instituto Universitario de Materiales de Alicante (IUMA), University of Alicante, Ap. 99, 03080, Alicante, Spain

^b^ Departamento de Química inorgánica and Instituto Universitario de Materiales de Alicante (IUMA), University of Alicante, Ap. 99, 03080, Alicante, Spain

**S1. EXPERIMENTAL**

**S1.1. Synthesis of gold nanoparticles.**

Solution 1: In a two-necked, round-bottom flask, 6.4 mg of the gold precursor (NaAuCl_4_∙2H_2_O) were dissolved in 5 mL of methanol. The resulting solution, light yellow in color, was stirred for 1 hour at room temperature.

Solution 2: in a two-necked, round-bottom flask, the protecting polymer (to get a 0.5 or 50 PVP/Au molar ratios), PVP was dissolved in 12 mL of ethylene glycol at 80ºC for 1 hour, under stirring conditions. Afterwards, solution 2 was cooled at 0ºC in an ice bath and solution 1 was added to solution 2, keeping stirring conditions. Immediately, pH of the resulting solution was adjusted to 9-10 with 0.5 mL of NaOH 1M under stirring conditions at 100ºC for 2 hours. After a few minutes, the color of the solution turned from yellow to dark red, indicating the reduction of the metal precursor to nanoparticles of zerovalent gold.

Gold nanoparticles (AuNPs) were purified using an excess of acetone to remove capping agent (PVP), solvents and remaining salts used in the synthesis; also, this procedure promotes the flocculation of the nanoparticles at the bottom of the vessel. The nanoparticles were subsequently dispersed in water to achieve a suspension with a concentration of 1 mg⋅mL^-1^.

**Step I-A**

**Step I-B**

**Step II**

**Step III**

**Step IV**

**Fig. S1**. Scheme of the gold-nanoparticles synthesis by the reduction-by-solvent method. (Step I) Preparation of solution 1: PVP-Ethylene glycol (Step I-A) and solution 2: NaAuCl_4_∙2H_2_O in methanol (Step I-B). (Step II) Cooling at 0ºC. (Step III) Mixing of solution 1 and 2, adjusting pH to 9-10 with NaOH 1M. (Step IV) Purification of AuNPs colloid with acetone.

**S1.2. Physicochemical characterization**

Temperature programmed desorption (TPD) experiments were performed in a DSC-TGA equipment (TA Instruments, SDT 2960 Simultaneous) coupled to a mass spectrometer (Thermostar, Balzers, GSD 300 T3) which was used to follow the m/z lines ascribed to the decomposition of surface functional groups from the surface of the carbon materials. The thermobalance was purged for 2 hours under a helium flow rate of 100 mL⋅min^-1^ and then heated up to 950°C (heating rate 20°C⋅min^-1^).

Scanning electron micrographs were taken using an ORIUS SC600 model Field Emission Scanning Electron Microscopy (FE-SEM) and a ZEISS microscope, Merlin VP Compact model, with and EDX Bruker, Quantax 400 model.

The textural properties of the materials have been evaluated by N_2_ adsorption isotherms at -196 °C in an automatic adsorption system (Autosorb-6, Quantachrome). Prior to the measurements, the samples were degassed at 250 °C for 4 h. Apparent surface areas have been determined by BET method (S_BET_) and total micropore volume (pores of size < 2 nm) has been assessed by applying the Dubinin-Radushkevich (DR) equation to the N_2_ adsorption isotherm.

**S2. RESULTS AND DISCUSSION**

**S2.1. Physicochemical characterization of carbon material precursor.**

Functionalization of MWCNT was studied by temperature programmed desorption (TPD) since this treatment incorporates oxygen functional groups in the surface which modifies the evolution of CO and CO_2_ during TPD. The CO_2_ evolution profile for pristine MWCNT shows, eventually, a negligible desorption of CO_2_ during heating and a small CO desorption, as shown in Fig. S2. However, the acid treatment of the carbon material induces the formation of surface oxygen groups which decompose as CO_2_ between 200-350ºC, 400-550ºC and 700-900ºC associated with carboxylic acid, anhydrides and lactones [1–3]. Likewise, the CO profile of MWCNT was affected by the treatment, showing desorption peaks at 400-650ºC and 600-900ºC related with phenol and carbonyl groups, respectively.

**A)**

**B)**

**Fig. S2. A-B)** TPD profiles for: A) CO_2_ and B) CO for pristine MWCNT and fMWCNT.

TEM images in Fig. S3 show the morphological features of pristine MWCNT and fMWCNT, respectively. On the one hand, the MWCNT in Fig. S3-A shows a typical tubular bamboo-like nanotubes shape, as a consequence of the synthesis procedure. On the other hand, modifications of the structure of the tubes, especially in the walls of the nanotubes in Fig. S3-B, such as broken-walls can be found as result of the oxidation reactions between the carbon material and the acid during the treatment.


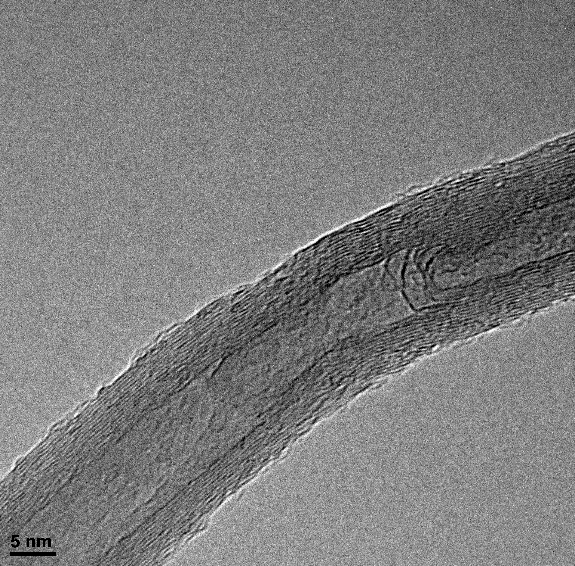

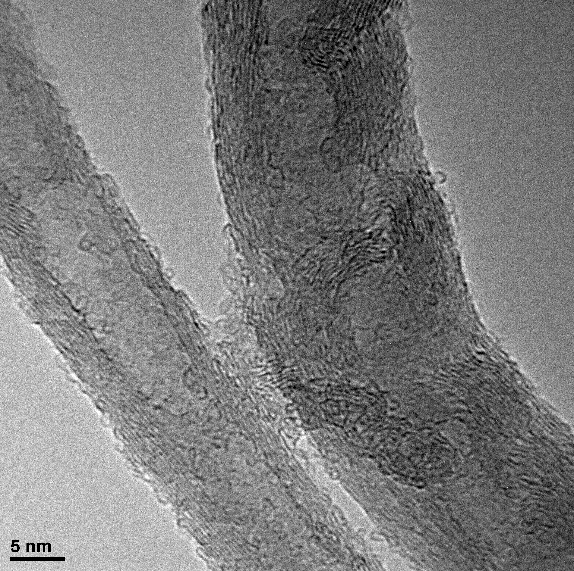


**Broken-walls**

**A)**

**B)**

**Fig. S3. A-B)** TEM images of: A) MWCNT and B) fMWCNT.

Additionally, these modifications in the nanostructure of the carbon material increase the specific surface of the material from 208 m^2^/g for MWCNT to 460 m^2^/g for fMWCNT as can be observed in the N_2_ 77K isotherms in Fig. S4 and Table S1. At the same time, most of the metal particles present in the pristine MWCNT are removed as a consequence of the treatment with nitric acid.

**Fig. S4**. N_2_ 77K isotherms for pristine MWCNT and fMWCNT in 3 M HNO_3_.

**Table S1**. Porous texture characterization results for carbon material precursors.

| Sample | S_BET_ / m^2^ g^-1^ | V_meso_ (N_2_) / cm^3^ g^-1^ |
| --- | --- | --- |
| MWCNT | 208 | 0.29 |
| fMWCNT | 460 | 0.57 |

**A)**

**B)**

**Fig. S5. A-B)** XPS spectra for Au 4f: A) fMWCNT-AuNPs-0.5 and B) fMWCNT-AuNPs-50 synthesized.

FE-SEM micrographs in Fig. S6 show the result of deposition of the different transducer materials synthesized, onto the surface of the glassy carbon electrode. Firstly, a homogeneous distribution of the carbon material modified with AuNPs is observed in Fig. S6-A and S6-C, covering the entire surface with no bundles or agglomeration of the nanotubes. Secondly, nanoparticles are distinguished of the carbon material using Back-Scattering electrons (BSE), (See Fig. S6-B and 6-D), showing a good distribution of the catalyst in the material and a small nanoparticle size in the synthesis with higher amount of PVP, as have been seen in the TEM images.


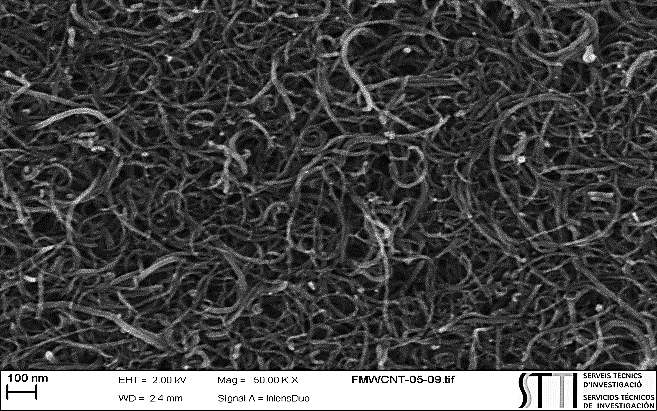

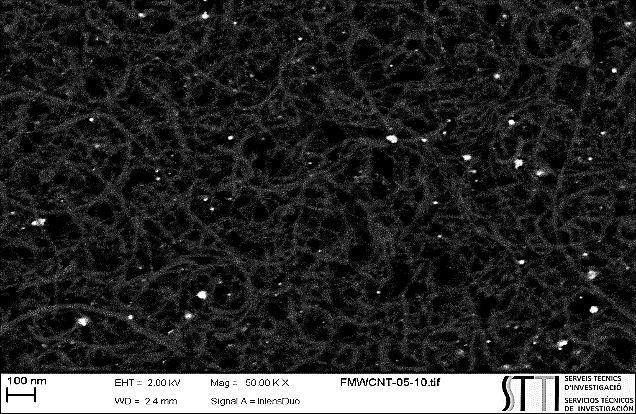

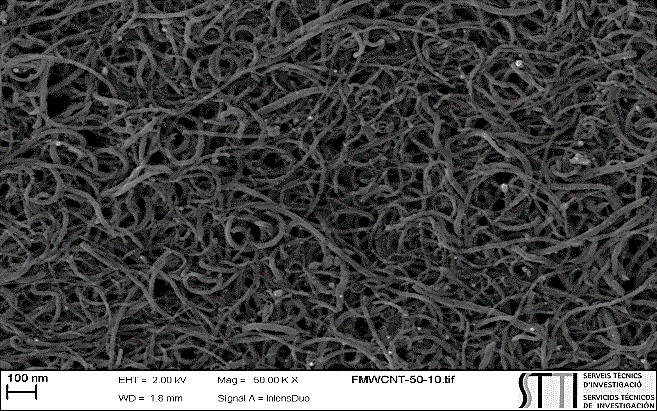

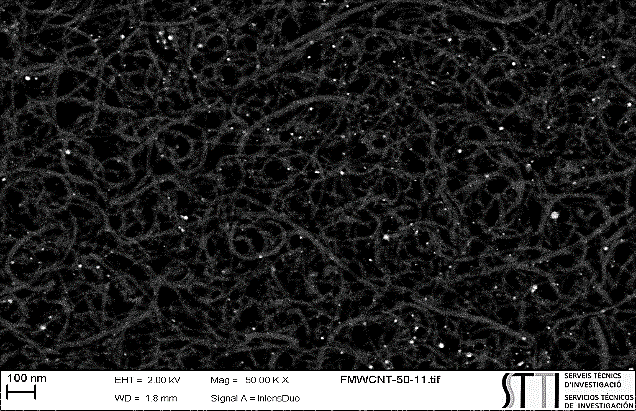


**A)**

**C)**

**B)**

**D)**

**Fig. S6. A-D)** FE-SEM micrographs for fMWCNT-AuNPs (with PVP/Au ratios of 0.5 and 50) onto glassy carbon surface: A) fMWCNT-AuNPs-0.5, B) BSE- fMWCNT-AuNPs-0.5, C) fMWCNT-AuNPs-50 and D) BSE-fMWCNT-AuNPs-50.

**S2.2.** **Electrochemical behavior of gold electrode modified with monoclonal antibodies to PSA.**

**B)**

**A)**

**Fig. S7**. CV for a polycrystalline gold electrode before and after the immobilization of monoclonal antibodies in 0.1M PBS (pH=7.2) at v_scan_ =50 mV⋅s^-1^ A) CV and B) Zoom area of gold oxide reduction process.

**Fig. S8**. CV for a polycrystalline gold electrode modified with monoclonal antibodies before and after cycling in 0.1M PBS (pH=7.2) at v_scan_ =50 mV⋅s^-1^.

**S2.3. Electrochemical performance of the different transducer materials in mediator.**

**B)**

**C)**

**A)**

**Fig. S9. A-C)** CV of the transducer material synthesized at different scan rates in 0.1M PBS + 0.5 mM Fc (pH=7.2) for A) fMWCNT, B) fMWCNT-AuNPs-0.5 and C) fMWCNT-AuNPs-50 from 10 mV⋅s^-1^ to 200 mV⋅s^-1^.

Analysis for the cathodic process show an increase of the peak current linearly in the range of scan rates of 10-200 mV⋅s^-1^ as can be seen in Fig. S10, suggesting a difussion-controlled electrochemical behavior for fWMCNT and fWMCNT-AuNPs [4]. Based on Randles-Sevicks´ equation (Eq. (1)):

*I_p_*=2.69x10^-5^∙*n*^3/2^∙*A*∙*D_0_*^1/2^∙*C_0_*^*^*v*_scan_^1/2^ (Eq. 1)

where *Ip* (A) the peak current, *n* is the number of exchanged electrons, *A* is the electroactive area (cm^2^), *D_0_* is the diffusion coefficient (cm^2^∙s^-1^), *C_0_^*^* is the concentration of the electroactive specie (mol∙cm^-3^), *v_scan_* is the scan rate (V∙s^-1^), slopes of the linear behavior might be associated with the term 2.69x10^-5^∙*n*^3/2^∙*A*∙*D_0_*^1/2^∙*C_0_*^*^, which suggested the electrochemical active area (A) in fMWCNT-AuNPs samples are higher and increase with the concentration of PVP in synthesis, given that the reduction of the nanoparticle size, as was mentioned above.

**Fig. S10**. Plot of specific current of cathodic peak vs. v_scan_^1/2^ in 0.1M PBS + 0.5 mM Fc (pH=7.2).

**SUPPORTING REFERENCE**

(1) Romanos, G. E.; Likodimos, V.; Marques, R. R. N.; Steriotis, T. A.; Papageorgiou, S. K.; Faria, J. L.; Figueiredo, J. L.; Silva, A. M. T.; Falaras, P. Controlling and Quantifying Oxygen Functionalities on Hydrothermally and Thermally Treated Single-Wall Carbon Nanotubes. *J. Phys. Chem. C* **2011**, *115* (17), 8534–8546.

(2) Brender, P.; Gadiou, R.; Rietsch, J.-C.; Fioux, P.; Dentzer, J.; Ponche, A.; Vix-Guterl, C. Characterization of Carbon Surface Chemistry by Combined Temperature Programmed Desorption with in Situ X-Ray Photoelectron Spectrometry and Temperature Programmed Desorption with Mass Spectrometry Analysis. *Anal. Chem.* **2012**, *84* (5), 2147–2153.

(3) Berenguer, R.; Marco-Lozar, J. P.; Quijada, C.; Cazorla-Amorós, D.; Morallón, E. Effect of Electrochemical Treatments on the Surface Chemistry of Activated Carbon. *Carbon N. Y.* **2009**, *47* (4), 1018–1027.

(4) Sanjuán, I.; Brotons, A.; Hernández-Ibáñez, N.; Foster, C. W.; Banks, C. E.; Iniesta, J. Boron-Doped Diamond Electrodes Explored for the Electroanalytical Detection of 7-Methylguanine and Applied for Its Sensing within Urine Samples. *Electrochim. Acta* **2016**, *197*, 167–178.
